# Age-related changes in the shell gland and duodenum in relation to shell quality and bone strength in commercial laying hen hybrids

**DOI:** 10.1186/s13028-019-0449-1

**Published:** 2019-03-12

**Authors:** Anna Wistedt, Yvonne Ridderstråle, Helena Wall, Lena Holm

**Affiliations:** 1 Department of Anatomy, Physiology and Biochemistry, Swedish University of Agricultural Sciences, Box 7011, 750 07 Uppsala, Sweden; 2 Department of Animal Nutrition and Management, Swedish University of Agricultural Sciences, Box 7024, 750 07 Uppsala, Sweden

**Keywords:** Bone strength, Carbonic anhydrase, Domestic hen, Eggshell formation, Eggshell quality, Oestrogen receptors

## Abstract

**Background:**

During the production period of laying hens, the number of cracked eggshells increases and the skeleton becomes brittle. Both these problems are related to ageing of the hen and cause economic problems for egg producers and impaired animal welfare. This study investigated key factors in the shell gland and duodenum related to eggshell quality and bone strength in laying hens during the production period. Five Lohmann Selected Leghorn (LSL) and five Lohmann Brown (LB), common hybrids in commercial egg production, were euthanized at 21, 29, 49 and 70 weeks (wk) of age. Blood samples for analysis of total calcium were taken at euthanization. Right femur and humerus were used for bone strength measurements and tissue samples from shell gland and duodenum were processed for morphology, immunohistochemical localisation of oestrogen receptors (ERα, ERβ), plasma membrane calcium ATPase (PMCA) and histochemical localisation of carbonic anhydrases (CA). Eggs were collected for shell quality measurements.

**Results:**

At age 49 week, shell and bone strength had both deteriorated, but the hens were then able to maintain the level until 70 week of age and femur bone strength even improved. The main physiological findings associated with the effects seen at 49 week were reduced gland density and a shift in balance between ERα and ERβ in the shell gland, which coincided with a reduction in CA activity in the duodenum. Somewhat surprisingly, capillary density and capillaries with CA activity both increased in the shell gland over time, the latter possibly mediated via ERβ. These findings were independent of hybrid. PMCA was found in both shell gland and duodenum, but appeared unrelated to the age-related changes in shell and bone quality.

**Conclusions:**

In hens around half-way through the production period, both shell quality and bone strength had deteriorated. Decreased gland density and a shift in the balance between ERα and ERβ in the shell gland, co-occurring with a dramatic drop in duodenal CA activity, are suggested as possible factors involved in age-related changes in shell and bone quality.

**Electronic supplementary material:**

The online version of this article (10.1186/s13028-019-0449-1) contains supplementary material, which is available to authorized users.

## Background

As laying hens age, they produce eggs with decreased shell quality, resulting in substantial economic losses due to cracked shells. The eggshell consists mainly of calcium carbonate (CaCO_3_) and the ionic precursors are supplied by the blood through trans-epithelial transport. During shell calcification, blood flow through the shell gland increases fivefold [1]. The acid–base balance in the shell gland is suggested to be an important factor during shell formation [2].

Eggshell calcium is derived from duodenal absorption, but skeletal stores are used as a secondary source. Duodenal calcium uptake increases sixfold during shell formation, but is affected negatively by age and declines from 37 weeks (wk) of age in layers [3]. Due to net efflux of calcium from the medullary bone of the skeleton, the hen becomes osteoporotic, which affects bone strength and causes animal welfare problems in the table egg industry [4–6].

Oestrogen production from the small follicles in the ovary starts at puberty and induces development of the oviduct and other female secondary sexual characteristics [7]. Oestrogen is important for calcium metabolism in the laying hen, acting on organs such as intestine, shell gland, skeleton and kidney and exogenous oestrogen boosts calcium uptake by the duodenum in laying hens [8]. According to previous research, there is no difference in circulating levels of oestrogen in plasma between egg-producing young and older hens, ranging from 35 to 100 week of age, but old non-laying hens have lower levels of oestrogen [9]. However, a recent study has shown that serum 17β-estradiol decreases in laying hens at 82 weeks, presumably due to an ageing ovary [10]. A reduction in oestrogen receptor ERα takes place in the shell gland towards the end of the laying season [11]. Besides ERα, ERβ is known to occur in birds [10, 12] and has recently been reported in the shell gland of two laying hen hybrids [13]. Expression of ERα seems to be predominant compared to ERβ in reproductive organs during sex differentiation in quail and in ovaries of adult domestic hens [14, 15]. In tissue expressing both receptors there are indication that ERα and ERβ have opposite functions, thus in mouse mammary gland epithelium ERα elicits and ERβ suppresses proliferation of the epithelium [16].

Eggshell carbonate is generally derived by hydration of metabolic CO_2_ to HCO_3_^−^, a reaction catalysed by carbonic anhydrases (CA). Inhibition of CA activity results in soft-shelled or shell-less eggs [17, 18] and CA activity in the shell gland is reported to decrease with age in laying hens [19]. CA are present in cell membranes of tubular glands and capillary endothelium in the shell gland, but it is not known whether their localisation alters during ageing. However, hens producing eggs with thinner shells have a reduced number of capillaries with CA activity [20, 21]. CA are also present in the duodenum, with duodenal CA activity being lower in hens laying soft-shelled eggs [22]. A possible functional relationship between CA and Mg^2+^HCO_3_^−^ ATPase may stimulate intestinal membrane transport of HCO_3_^−^ and other ions as described in rats [23]. It is not known whether there are any age-related changes in duodenal CA localisation.

Thus, cellular mechanisms in both shell gland and duodenum are important in providing the Ca^2+^ and HCO_3_^−^ needed for proper shell formation and skeletal health in laying hens. The aim of the present study was to investigate ERα, ERβ, plasma membrane calcium ATPase (PMCA) and CA in both shell gland and duodenum during the whole laying period, in parallel with measurements of eggshell quality and bone strength.

## Methods

### Animals, housing and management routines

Laying hens of two different hybrids, Lohmann Selected Leghorn (LSL, n = 1260) and Lohmann Brown (LB; n = 1320) (Gimranäs AB, Sweden), arrived at the poultry house at 15 week of age. The birds were housed in the university poultry research facility under conditions similar to those in commercial egg production. The birds were kept in furnished 8-hen cages, as described by Wall and Tauson [24] and the light regime was gradually increased from 9 h/24 h to 14 h/24 h by 23 week of age. All hens were fed ad libitum according to a phase feeding programme for commercial hens distributed by a Swedish feed manufacturer (Lantmännen) and had free access to water. A random sample of eight cages was picked and five hens of each hybrid were housed per cage. The birds in these cages were considered focal animals in the study. Samples were taken from hens when they were 21 week, 29 week, 49 week and 70 week, to reflect four different laying stages, i.e. early laying, peak laying, intermediate laying and late laying, respectively. Ten focal laying hens of each age from two cages (five LSL and five LB) were sacrificed, i.e. a total of 20 LSL hens and 20 LB hens were sampled. Egg production was monitored in the entire poultry house throughout the production period and was used as a reference for production parameters. The eggs were collected each morning and number of eggs and egg weight were recorded weekly. Rate of lay (%) was calculated as number of eggs laid (per day and hen) × 100. The cages complied with Swedish Animal Welfare Directives and the study was approved by the Uppsala Local Ethics Committee.

### Eggshell measurements

Eggs from focal laying hens in full production (29 week, 49 week and 70 week) were considered representative of the age of the whole flock and were collected for shell quality measurements on 5 consecutive days during 1 week prior to sacrifice. Shell deformation, shell breaking strength, shell thickness and shell weight with and without shell membranes were recorded. For a detailed description of eggshell measurements, see Additional file 1.

### Tissue preparation

The hens were killed by intravenous injection of pentobarbital sodium (100 mg/mL, Apoteket AB, Umeå, Sweden) into the wing vein and body weight was recorded. Left oviduct and the small intestine were rapidly removed. The oviduct was dissected free from the mesoviductus and straightened, the length was measured from the vaginal orifice to the fimbriated infundibulum and the localisation of the egg was recorded. The shell gland was then cut open lengthwise and pieces cut from the middle part were removed for fixation. A 2 cm long piece of duodenum was removed immediately distal to the duodenal loop and cut open. All tissues were divided in two and pinned to small rectangles of cork to minimize tissue distortion. One piece of each tissue was fixed in 2.5% glutaraldehyde in 0.067 M phosphate buffer (pH 7.2) for CA histochemistry and the other piece was fixed in 4% paraformaldehyde in 0.067 M phosphate buffer (pH 7.2) for immunohistochemistry, both for 24 h at 4 °C. After rinsing in phosphate buffer, the tissue was trimmed into 2 mm thick transverse slices. Following dehydration in increasing concentrations of ethanol, samples were embedded in a water-soluble resin (Leica Historesin, Heidelberg, Germany) for CA histochemistry and in paraffin for immunohistochemistry.

### Bone strength of femur and humerus

The right leg and wing bones were obtained at euthanization, frozen and stored until analysis. Before measurement, the femur and humerus were thawed to room temperature and dissected free from skin, ligaments and muscles. The bones were tested to breaking point on a three-point bending electromechanical testing machine (Avalon Technologies, Rochester, MN, USA). The loading speed was 1 mm/s and the span length used was 30 mm. Bone strength of femur and humerus was tested by three-point bending at the mid-diaphyseal region of the bone and the load at failure was measured. Digital data were collected 50 times per second until failure, using software provided with the testing machine (Testware II).

### Total calcium in plasma

Blood samples were collected in heparinised tubes immediately prior to euthanization. The samples were centrifuged at 3000 rpm (1400*g*) at 4 °C for 10 min to separate the plasma. Plasma samples were stored at − 70 °C until analysis of total calcium (mmol/L) was performed (Calcium Architect cSystems, Aeroset System, Abbott Laboratories, Solna, Sweden).

### CA histochemistry

Histochemical localisation of CA activity was detected according to Ridderstråle’s histochemical method [25], resulting in a black precipitate at sites of active CA. The specificity of the staining was checked using the CA inhibitor acetazolamide. For details of the histochemical staining procedure and preparation, see Additional file 1.

### Immunohistochemistry

ERα was detected using a rabbit anti-ERα (clone 60C, Millipore, USA), ERβ was detected using a mouse monoclonal antibody (MCA 1974ST, Serotec, Düsseldorf, Germany) and PMCA was detected using a mouse monoclonal antibody (5F10 ab2825, Abcam 330, Cambridge Science Park, Cambridge, CB4 0FL, UK). For details of the immunohistochemical staining and preparation, see Additional file 1.

### Image analysis and morphometric evaluation

The birds were culled on different time-points, but morphometric evaluation was done with all age groups at the same time. One person (AW) carried out all the morphometric measurements. All slides were coded and the examination was performed blind. Only areas free from artefacts were chosen for evaluation. Digital images of CA-stained sections from the shell gland were taken with a Nikon Microphot-FXA microscope using a 10× objective lens and for mucosal height of duodenum using a 4× objective. For details of CA activity and histological evaluation, see Additional file 1.

For the immunohistochemical evaluation of ERα and ERβ in the shell gland and of PMCA in the duodenum, the localisation was described and the staining of each structure on one slide per bird was scored for intensity on a scale from 0 to 3, where zero corresponds to no staining and three represents strong staining. On the same occasion, the frequency of tubular glands in the mucosal fold of the shell gland was scored as dense or not dense.

No differences were observed regarding the immunohistochemical staining of PMCA in the shell gland and the staining of ERα and ERβ in the duodenum, and these sections were therefore not subjected to further measurements.

### Statistical analysis

Variables such as body weight and oviduct length, bone measurements, plasma calcium, egg production and shell quality, and morphometric measurements such as number of capillaries and mucosal height, were analysed statistically by the mixed procedure (Proc Mixed) in SAS^®^ (SAS Institute Inc., Cary, NC, USA, version 9.4) and applying Bonferroni correction for multiple comparisons. Data from the entire poultry house (with 61 cages) were used as reference and analysis of production [rate of lay (%) and egg weight] included the fixed effects of age (n = 57 weeks) and hybrid (n = 2). All other statistical models included the fixed effects of hybrid (n = 2), age (n = 3 or 4) and interaction between hybrid and age and the random effect of sampling day nested within the interaction between hybrid and age. In the analysis of production, each group, i.e. hens housed in the same cage, was treated as one experimental unit. For egg shell quality traits each egg was one experimental unit. Egg weight was included as a covariate in the analysis of shell deformation test, shell breaking strength and shell weight. In the analysis of body weight, organ measurements, total calcium and bone strength, each hen was treated as an experimental unit, i.e. five replicates per hybrid and age. Body weight was included as a covariate in the analysis of bone strength and total calcium.

Morphometric staining intensity, graded from 0 (no staining) to 3 (strong staining), and density of tubular glands (less dense = 1, dense = 2) were analysed by Likelihood Ratio Chi Square test using SAS^®^. The statistical models included the fixed effects of hybrid (n = 2) and age (n = 4). Each hen was treated as an experimental unit, i.e. five replicates per hybrid and age. Differences were considered significant at P < 0.05.

## Results

### Body weight and organ measurements

Body weight (mean ± standard error, n = 20) was 2037 ± 62.2 g for LB hens and 1598 ± 37.9 g for LSL hens (P < 0.0001). Body weight of all layers was affected by age, but there was no interaction between age and hybrid (Table 1).

**Table 1:** Body weight, oviduct length, breaking strength of femur, breaking strength of humerus and total calcium in plasma of two layer hybrids

	LSL and LB						Statistical significance (P value)		
	21 weeks (n = 10)	29 weeks (n = 10)	49 weeks (n = 10)	70 weeks (n = 10)	LB (n = 20)	LSL (n = 20)	Age	Hybrid	Age* Hybrid
Body weight (g)	1661 ± 56.7^c^	1726 ± 80.4^bc^	1985 ± 123.2^a^	1898 ± 107.1^ab^	2037 ± 62.2^a^	1598 ± 37.9^b^	0.002	< 0.0001	0.2
Oviduct length (cm)^d^	54 ± 0.8^b^	56 ± 0.8^b^	62 ± 1.2^a^	63 ± 1.5^a^	60 ± 1.2	58 ± 1.2	< 0.0001	0.2	0.6
Breaking strength, femur (N)^e^	218 ± 10.4^a^	168 ± 9.7^bc^	137 ± 10.5^b^	182 ± 9.7^ac^	162 ± 9.1	191 ± 9.1	0.0001	0.07	0.04
Breaking strength, humerus (N)^e^	252 ± 10.3^a^	237 ± 9.7^ab^	198 ± 10.4^b^	225 ± 9.6^ab^	235 ± 9.0	220 ± 9.0	0.02	0.3	0.1
Total calcium (mmol/L)	5.5 ± 0.14^b^	6.1 ± 0.24^ab^	6.5 ± 0.43^ab^	7.0 ± 0.42^a^	6.0 ± 0.25	6.6. ± 0.26	0.02	0.1	0.1

Lohmann Selected Leghorn (LSL) and Lohmann Brown (LB) at four different ages (21, 29, 49, 70 weeks) during the production period. Mean ± standard error. Values without a superscript or with the same letter (^a^, ^b^ or ^c^) do not differ significantly (P > 0.05)

^d^Measured from the orifice to the fimbriated infundibulum

^e^Measured by three-point bending, load (Newton) at failure; body weight included as a covariate in the analysis

All hens had fully developed follicles in the ovary (data not shown) and a well-developed left oviduct, the length of which increased from 21 to 70 week of age (Table 1). The length of the oviduct did not differ between hybrids and there was no interaction between age and hybrid. The localisation of the egg in the oviduct was not correlated with any of the other parameters measured.

### Bone strength of femur and humerus

Bone strength of femur decreased from week 21 and continued to decrease until 49 week of age. A small recovery was seen at 70 week (Table 1). There was no difference between the two hybrids, but there was an interaction between hybrid and age. Bone strength of humerus decreased from 21 to 49 week and remained constant thereafter (Table 1). There was no difference between the two hybrids and no interaction between hybrid and age.

### Total calcium in plasma

Total calcium concentration in plasma increased from early lay to end of lay (Table 1). There was no difference between the two hybrids and no interaction between hybrid and age.

### Egg production and shell quality

**Fig. 1:**
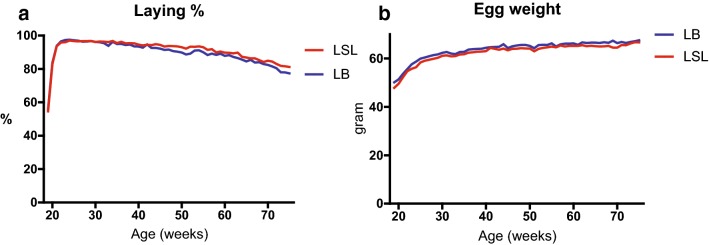
Production data (means) for the entire poultry house in weeks 1–57 in the production period of the laying hens. Lohmann Brown (LB) (n = 61) cages and Lohmann Selected Leghorn (LSL) (n = 61) cages. **a** Rate of lay (%), number of eggs laid/day and hen ×100 [significantly higher for LSL hens (P < 0.0001)]. **b** Egg weight in grams [significantly higher for LB hens (P < 0.0001)]

In the reference group, rate of lay was affected by age and decreased towards the end of the production period (P < 0.001) and was higher for the LSL hens than the LB hens (P < 0.001) (Fig. 1a). Egg weight was affected by age and increased towards the end of the production period (P < 0.001) and the LB eggs were heavier than the LSL eggs (P < 0.001) (Fig. 1b).

In the eggs from focal laying hens, shell deformation increased from 29 to 49 week and remained constant thereafter until 70 week of age (Table 2), with no difference between the two hybrids and no interaction between hybrid and age. Breaking strength was decreasing from 29 to 49 week and remaining constant thereafter to 70 week of age. Breaking strength was affected by hybrid, with LB eggs having higher breaking strength than LSL eggs (Table 2). There was no interaction between hybrid and age. Shell weight was affected by age, decreasing from 29 to 49 week of age and then tending to increase from 49 to 70 week of age (P = 0.086) (Table 2). There was no difference between the two hybrids and no interaction between hybrid and age. Eggshell thickness including shell membrane was affected by age, the thickness decreased from 29 week and 49 week to 70 week of age, no difference between hybrid or interaction between age and hybrid (Table 2). Eggshell thickness excluding shell membrane was affected by age, as it decreased from 29 to 49 week, then recovered and increased between 49 and 70 week of age (Table 2). Eggshell thickness excluding shell membrane was not affected by hybrid or interaction between age and hybrid.

**Table 2:** Eggshell quality measurements from two layer hybrids

	LSL and LB					Statistical significance (P value)		
	29 week (n = 46)	49 week (n = 46)	70 week (n = 40)	LB (n = 62)	LSL (n = 70)	Age	Hybrid	Age* Hybrid
Shell deformation test (µm)^c^	60 ± 1.2^b^	76 ± 2.3^a^	80 ± 1.9^a^	70 ± 1.6	73 ± 1.5	0.0003	0.5	0.4
Shell breaking strength (g)^c^	4678 ± 91^a^	4098 ± 91^b^	4122 ± 124^b^	4465 ± 85^a^	4171 ± 77^b^	0.0001	0.02	0.2
Shell weight (g)^c^	5.70 ± 0.06^a^	5.58 ± 0.07^b^	5.85 ± 0.09^ab^	5.76 ± 0.06	5.67 ± 0.05	0.02	0.3	0.8
Shell thickness incl. membrane (mm)^d^	0.392 ± 0.0039^a^	0.380 ± 0.0048^a^	0.373 ± 0.0092^b^	0.389 ± 0.0038	0.383 ± 0.0034	0.0003	0.9	0.8
Shell thickness excl. membrane (mm)^d^	0.360 ± 0.0039^a^	0.346 ± 0.0050^b^	0.349 ± 0.0089^ab^	0.360 ± 0.0039	0.352 ± 0.0035	0.03	0.5	0.3

Lohmann Selected Leghorn (LSL) and Lohmann Brown (LB) at three different ages (21, 49, 70 weeks) during the production period. Mean ± standard error. Values without a superscript or with the same letter (^a^ or ^b^) do not differ significantly (P > 0.05)

^c^Egg weight included as a covariate in analysis of shell deformation, shell breaking strength and shell weight

^d^Shell weight included as a covariate in analysis of shell thickness including and excluding membrane

### Morphometric measurements

#### Shell gland

The total number of shell gland capillaries/mm^2^ at the top of the mucosal fold was affected by age, increasing from 21 to 29 week of age and remaining constant thereafter (Table 3). There was no difference between hybrids and no interaction between age and hybrid.

**Table 3:** Morphology measurements of shell gland and duodenum from two layer hybrids

	LSL and LB						Statistical significance (P value)		
	21 week (n = 10)	29 week (n = 10)	49 week (n = 10)	70 week (n = 10)	LB (n = 20)	LSL (n = 20)	Age	Hybrid	Age* Hybrid
Number of capillaries/mm^2^ in shell gland	289 ± 13.4^b^	370 ± 20.4^a^	332 ± 19.3^ab^	358 ± 20.8^ab^	322 ± 13,8	351 ± 14.9	0.02	0.2	0.2
Number of CA- positive capillaries/mm^2^ in shell gland	0.1 ± 0.06^c^	0.4 ± 0.25^bc^	10.9 ± 5.08^ab^	22.4 ± 9.03^a^	8.2 ± 4.65	8.7 ± 3.56	0.002	0.5	0.9
Mucosal height in the duodenum (µm)^d^	1387 ± 65.9	1376 ± 44.9	1582 ± 93.1	1436 ± 67.1	1569 ± 53.6^a^	1322 ± 29.2^b^	0.03	< 0.0001	0.006
Depth of crypt of Lieberkühn in duodenum (µm)^e^	153 ± 7.0	145 ± 8.0	132 ± 8.0	137 ± 7.8	142 ± 4.7	141 ± 6.4	0.3	0.9	0.4

Lohmann Selected Leghorn (LSL) and Lohmann Brown (LB), at four different ages (21, 29, 49, 70 weeks) during the production period. Mean ± standard error. *CA* carbonic anhydrase. Values without a superscript or with the same letter (^a^, ^b^ or ^c^) do not differ significantly (P > 0.05)

^d^Measured from muscularis mucosa to top of the villi

^e^Measured from muscularis mucosa to base of villi

**Fig. 2:**
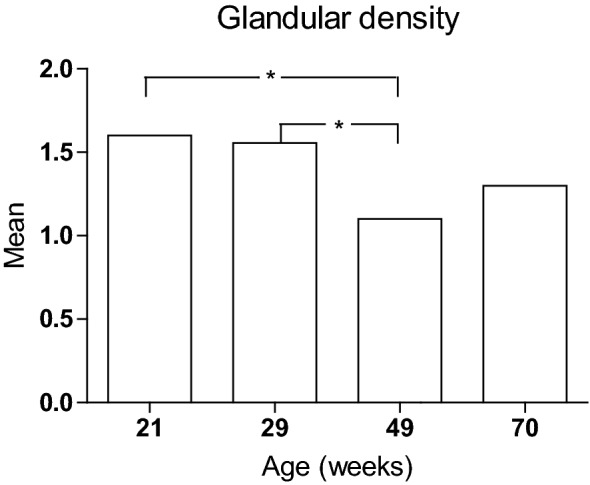
Density of tubular glands in the shell gland of Lohmann Selected Leghorn (LSL) and Lohmann Brown (LB) hens at different ages during the production period. Morphometric density of tubular glands (less dense = 1) and (dense = 2) analysed by Likelihood Ratio Chi Square test using SAS^®^. The statistical models included the fixed effects of hybrid (n = 2) and age (n = 4)

The density of tubular glands in the shell gland was lowest at 49 week of age, as revealed by a Chi square test. The glands were more dense at 21 week compared with 49 week (P = 0.01) and more dense at 29 week compared with 49 week of age (P = 0.03) (Fig. 2). No difference in density of tubular glands was found between 21 and 70 week, between 29 and 70 week or between 49 and 70 week of age (Fig. 2), and there was no difference between the hybrids.

#### Duodenum

**Fig. 3:**
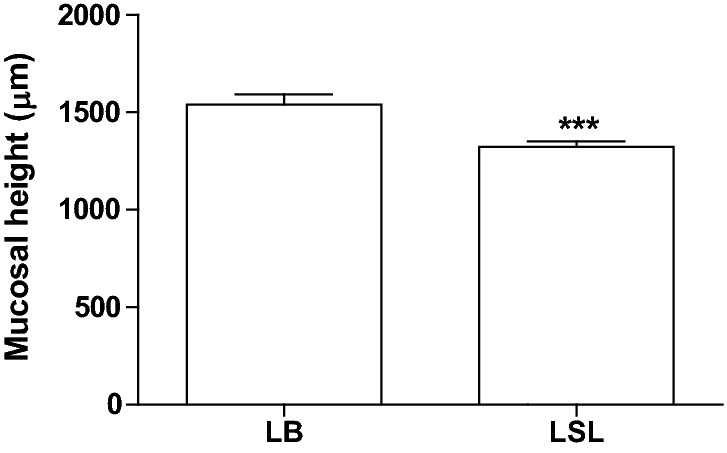
Mucosal height in the duodenum of Lohmann Selected Leghorn (LSL) and Lohmann Brown (LB) hens at different ages during the production period. The height of mucosa in the duodenum was measured in five fields/section, measured from muscularis mucosa to top of the villi, and a mean was calculated for each bird

The mucosal height was higher in LB hens than in LSL hens (Fig. 3, Table 3). The mucosal height of the LB hens increased from 29 to 49 week of age (P = 0.004), while the mucosal height in LSL hens was unaffected by age (Table 3). The depth of crypts of Lieberkühn in the duodenum was not affected by age and there was no difference between hybrids and no interaction between hybrid and age (Table 3).

### CA histochemistry

Sections incubated for CA show black staining at sites of active enzyme. As controls one section each of all samples from shell gland and duodenum were incubated with the CA inhibitor acetazolamide added to the incubation medium. None of these sections contained any significant black staining, indicating that staining seen after incubation is CA.

#### Shell gland

**Fig. 4:**
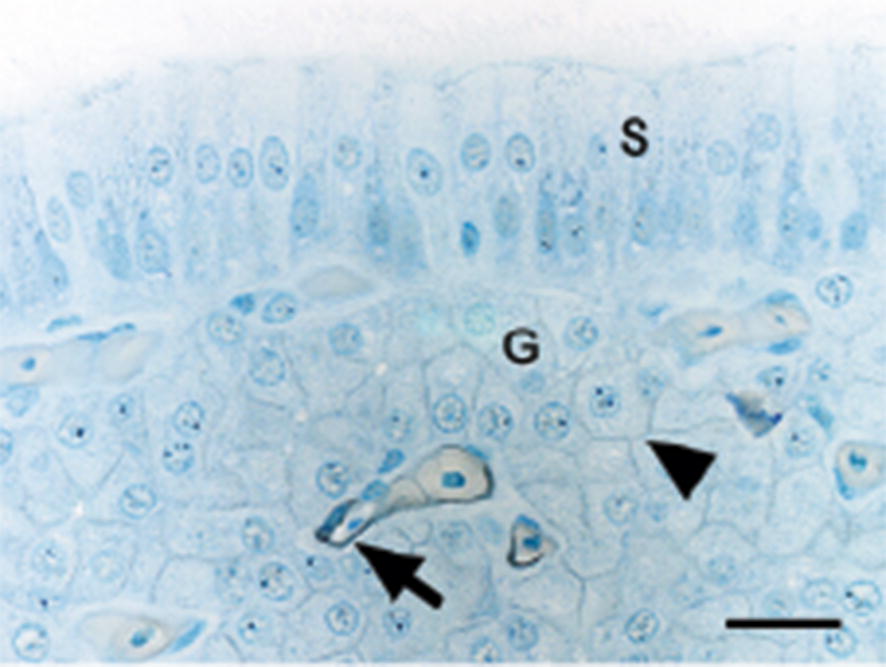
Localisation of active carbonic anhydrase (CA) in shell gland of Lohmann Selected Leghorn (LSL) and Lohmann Brown (LB) hens at different ages during a production period. Active CA is shown as black staining. Shell gland mucosal fold of 70-week-old LSL hens shows no detectable staining for CA in surface epithelium (S). Intense membrane-bound staining of endothelial cells (arrow) in capillaries and weak membrane-bound staining of tubular glands (G and arrowhead) can be seen. Bar = 20 µm. Weak azure blue counterstain

The surface epithelium was unstained in all hens, regardless of age or hybrid (Fig. 4). A minor proportion of the tubular glands showed weak membrane-bound staining for CA activity, which was present at all ages except 29 week and in the LB hens was absent also at 70 week (Fig. 4).

The capillary endothelium showed intense membrane staining. The numbers of CA positively stained capillaries increased with increasing age and were situated at the top of the shell gland mucosal folds (Fig. 4, Table 3). There was no difference between hybrids and no interaction between age and hybrid.

#### Duodenum

Surface epithelium of villi showed intense staining for CA activity in the lateral cell membranes and brush border. Cytosolic staining was moderate. The staining of surface epithelial cells gradually decreased towards the top of the villi. Capillaries with intense membrane-bound staining were found in villi lamina propria, crypt region and muscularis mucosa. In the crypts of Lieberkühn, lateral cell membranes showed intense staining and the cytosol weak to moderate staining. In the cells of muscularis, membrane-bound staining was moderate.

**Fig. 5:**
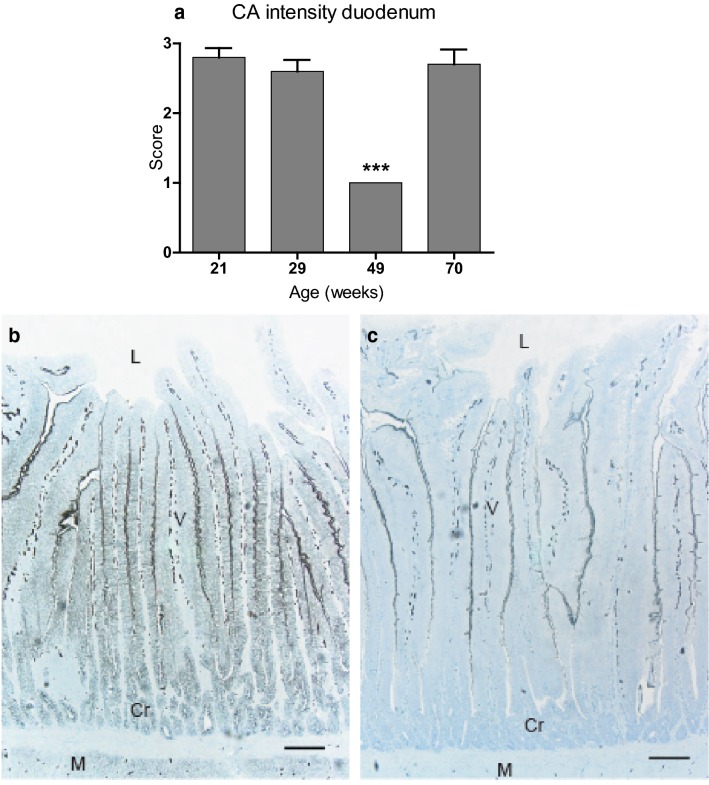
Intensity scoring of carbonic anhydrase (CA) activity in duodenum of Lohmann Selected Leghorn (LSL) and Lohmann Brown (LB) hens (n = 10) at different ages during a production period. **a** Intensity scoring of carbonic anhydrase (CA) activity in duodenum. Score 1: weak or absent staining. Score 2: intermediate staining. Score 3: strong staining. Mean ± standard error. ***P < 0.001. **b** Duodenum of 21-week-old LB hen (score 3). Surface epithelium of villi (V) shows intense staining for CA activity in lateral cell membranes and brush border, and moderate cytosolic staining. Note capillaries with intense membrane-bound staining of endothelial cells in the lamina propria of villi. In the crypts of Lieberkühn (Cr), lateral cell membranes are intensely stained and the cytosol weak to moderately stained. In the muscularis (M), membrane-bound staining is moderate. Lumen (L). **c** Duodenum of 49-week-old LB hen (score 1). Surface epithelium of villi (V) shows none or very weak staining for CA activity in lateral cell membranes and moderate staining of brush border. Cytosolic staining is very weak or absent. Note capillaries with intense membrane-bound staining of endothelial cells in lamina propria of villi. No staining of cell membranes or cytosol can be seen in crypts of Lieberkühn (Cr) or smooth muscle cells of circular muscularis (M). Lumen (L), bar = 200 µm. Weak azure blue counterstain in both images

In general, the scoring of staining intensity for CA activity was strong or moderate in all age groups except at 49 week of age, when staining was very weak or absent (Fig. 5a–c).

### Oestrogen receptor α

Immunohistochemical localisation of ERα in the epididymis of roosters was used as a positive control and the results were in agreement with previous findings [26]. The negative controls showed no significant staining.

#### Shell gland

**Fig. 6:**
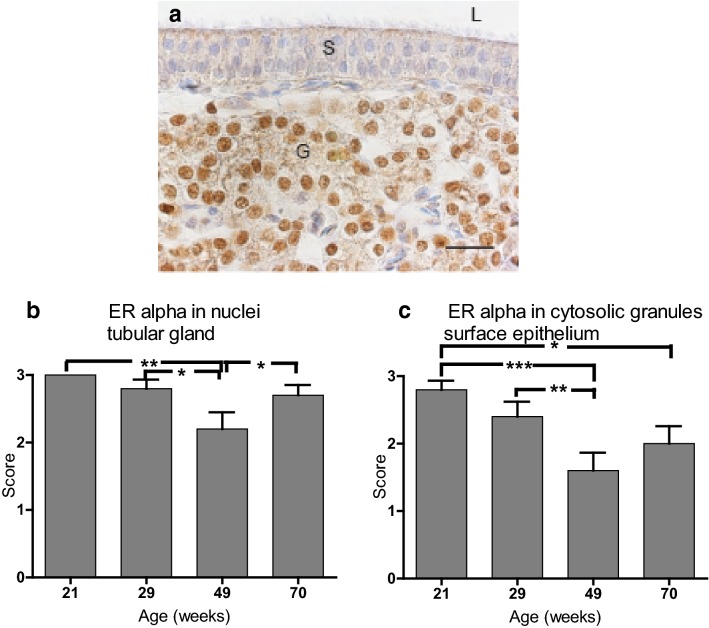
Oestrogen receptor α (ERα) immunolocalization in shell gland of Lohmann Selected Leghorn (LSL) and Lohmann Brown (LB) hens at different ages during a production period. **a** Shell gland mucosal fold of 29-week-old LSL hen showing strong staining of nuclei in tubular gland cells (G), moderate cytosolic granular staining for ERα in surface epithelium (S) and unstained nuclei of both ciliated cells and non-ciliated cells. Lumen (L), bar = 20 µm, weak haematoxylin counterstain. **b**, **c** Intensity scoring of oestrogen receptor α (ERα) in shell gland mucosal fold of LB and LSL hens (n = 10) at four different ages during a production period. Score 0: no staining. Score 1: weak staining. Score 2: intermediate staining. Score 3: strong staining. **b** ERα staining in nuclei of tubular glands. **c** ERα staining of cytosolic granules in surface epithelial cells. Mean ± standard error. *P < 0.05, **P < 0.01, ***P < 0.001

Strong staining for ERα was found in the nuclei and weak to moderate cytosolic staining in tubular gland cells. The nuclei of the surface epithelium cells were unstained, but weak to moderate cytosolic granular staining was found in the apical part of both non-ciliated and ciliated cells (Fig. 6a). The intensity decreased with age in nuclei in tubular glands (Fig. 6b) and in the cytosolic granules of surface epithelial cells (Fig. 6c). There was no detectable difference between hybrids and no interaction between staining and hybrid or age.

The endothelium of capillaries tested negative for ERα, but in larger blood vessels the endothelium contained both stained and unstained nuclei. The nuclei in the erythrocytes were negative. Smooth muscle cells of muscularis mucosa and blood vessels had moderate to weak nuclear staining for ERα. There was no effect of age or hybrid and no interaction between hybrid and age.

#### Duodenum

In duodenum, only weak staining for ERα was found in the nuclei of smooth muscle cells of muscularis mucosa and there were no detectable differences in staining intensity or localization of ERα between hybrids and age and no interactions between hybrid and age. No staining of ERα was found in surface epithelial cells of villi or crypts of Lieberkühn.

### Oestrogen receptor β

Testis and epididymis from rooster and ovary and uterus from pig were used as positive control tissues. The results were in agreement with previous findings [26, 27]. The negative controls showed no significant staining.

#### Shell gland

**Fig. 7:**
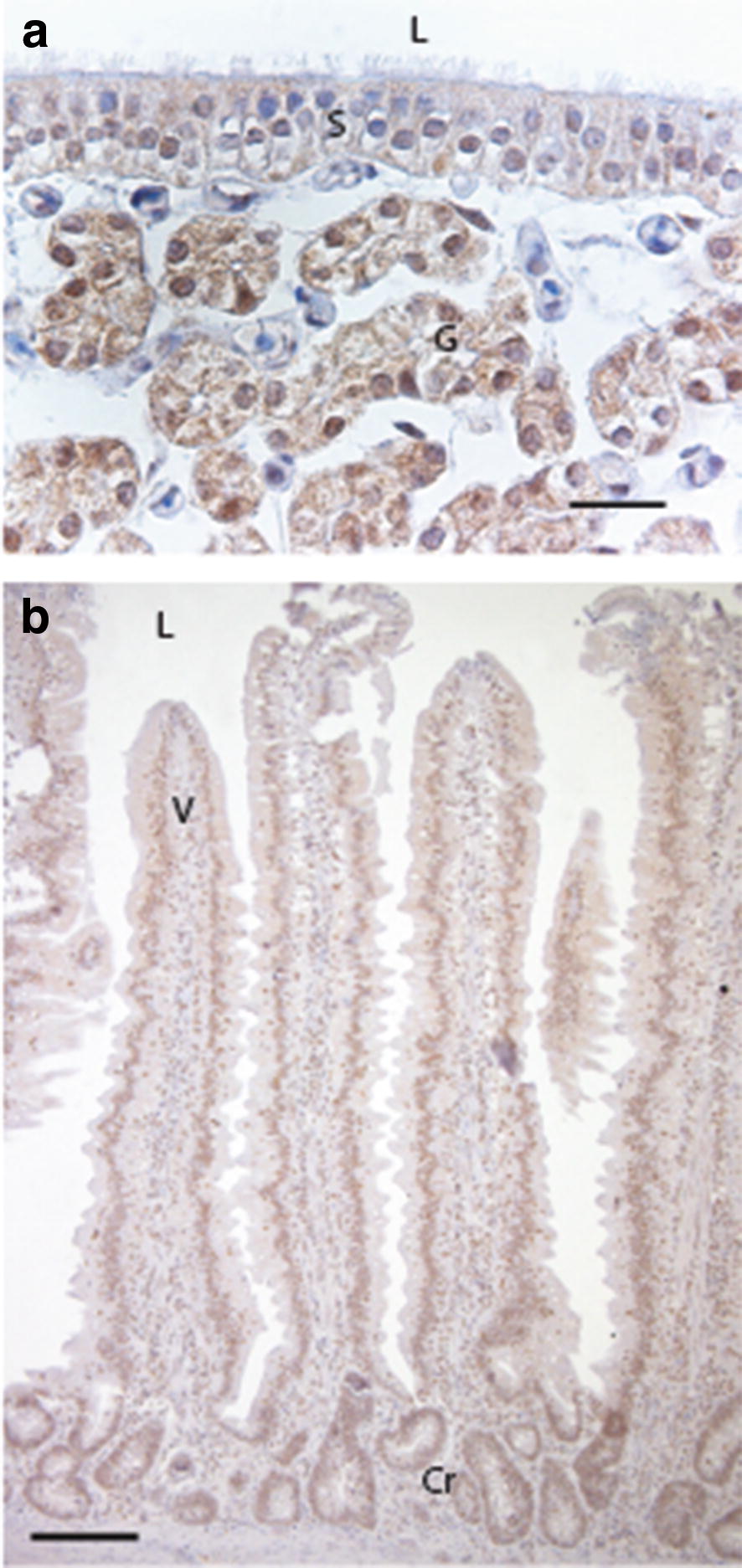
Oestrogen receptor β (ERβ) immunolocalization in shell gland and duodenum of Lohmann Selected Leghorn (LSL) and Lohmann Brown (LB) hens at different ages during a production period. **a** Shell gland mucosal fold of 49-week-old LSL hen. Strong nuclear staining for ERβ in non-ciliated cells of the surface epithelium (S). Weakly stained or negative nuclei in ciliated cells. Tubular gland cells (G) show strong nuclear staining for ERβ. Weak to moderate cytosolic staining in surface epithelium (S) and tubular gland cells (G). Lumen (L), bar = 20 µm. **b** Duodenum of 21-week-old LSL hen showing moderate to weak nuclear staining for ERβ in surface epithelium of villi (V), crypts of Lieberkühn (Cr) and smooth muscle cells (M). Weak cytosolic staining for ERβ in the surface epithelium (S). The staining gradually decreases towards the top of the villi and most of the nuclei at the top are negative. Lumen (L), bar = 100 µm. Weak haematoxylin counterstain in both images

The strongest staining for ERβ was found in the nuclei of the non-ciliated cells in the surface epithelium. Weak to moderate staining was present in the nuclei of some ciliated cells, while others were negative (Fig. 7a). Cytosolic staining for ERβ was found in the surface epithelium.

The tubular gland cells showed strong nuclear staining and weak cytosolic staining. The cytosolic staining increased from 21 to 29 week of age (P = 0.04). Then the staining intensity decreased from 29 to 49 week of age (P = 0.004). The staining intensity was also significantly weaker at 70 week of age compared to 29 week of age (P = 0.047). For details of immunohistochemical staining intensity, see Additional file 2.

Strong to moderate staining was also found in connective tissue cells. The endothelium of both capillaries and larger blood vessels showed strong nuclear staining. Nuclear staining of erythrocytes was absent, but nuclei in leukocytes were occasionally stained for ERβ. Smooth muscle cells of muscularis had strong to moderate nuclear staining, as did smooth muscle cells of blood vessels.

There was no detectable difference in intensity or localisation of ERβ between the hybrids.

#### Duodenum

In duodenum, moderate to weak staining for ERβ was found in the nuclei of surface epithelium, crypts of Lieberkühn and smooth muscle cells (Fig. 7b). The staining in the villi gradually decreased towards the top and most nuclei at the top were negative. There was weak cytosolic staining for ERβ in the surface epithelium. There were no detectable differences in intensity or localisation of ERβ between the hybrids or ages.

### PMCA

#### Shell gland

**Fig. 8:**
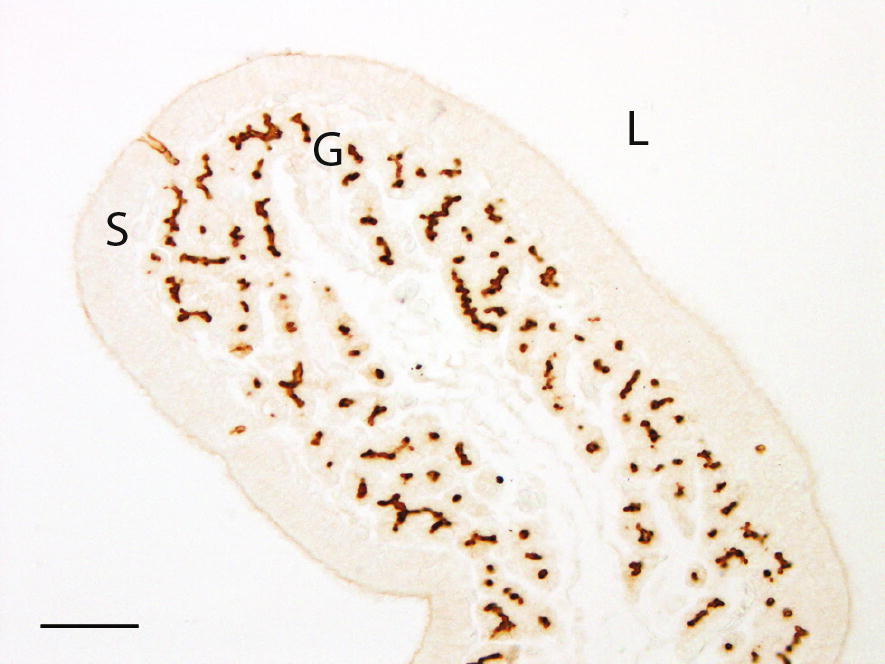
Plasma membrane Ca^2+^ ATPase (PMCA) immunolocalisation in shell gland of Lohmann Selected Leghorn (LSL) and Lohmann Brown (LB) hens at different ages during a production period. Shell gland mucosal fold of 29-week-old LB hen. Strong staining in the apical membrane of the tubular gland cells (G), weak staining apical in the surface epithelium (S). Lumen (L), bar = 50 µm

Strong staining for PMCA was found in the apical membrane of the tubular gland cells and weak staining was found apically in the surface epithelial cells (Fig. 8). There were no detectable differences in intensity or localisation of PMCA staining between hybrids or ages. The negative controls showed no significant staining.

#### Duodenum

In duodenum, staining for PMCA was found in the basolateral membranes of the surface epithelial cells. The staining intensity was strongest at the top of the villi and gradually decreased towards the base. The staining at the base of the villi increased with age in the LB hybrids (P = 0.009), but only tended to decrease with age in the LSL hybrids (P = 0.066). There was no detectable difference in staining intensity between hybrids and no interaction between staining in the basolateral membrane of the surface epithelial cells and hybrid or age. Weak basolateral membrane-bound staining was found in the crypts of Lieberkühn cells, with the staining intensity being stronger in the LSL hybrids than in the LB hybrids (P = 0.04). This staining was not affected by age. The negative controls showed no significant staining.

For details of immunohistochemical staining intensity, see Additional file 2.

## Discussion

The main findings in this study were that shell quality and bone strength were both negatively affected about midway through the production period, at 49 week of age. However, the hens seemed to be able to maintain this level of shell quality, while bone strength even improved later in the production period. At 49 week of age, we also detected a dramatic reduction in duodenal CA activity, coinciding with decreased gland density in the shell gland and a shift in the balance between ERα and ERβ in the shell gland. PMCA was found in both shell gland and duodenum, but appeared to be independent of the age-related changes in shell and bone quality.

Rate of lay decreased, as expected, with age in both hybrids, while egg weight increased from 29 to 49 week of age and then remained stable until 70 week. Shell quality followed a similar pattern, showing a significant decline regarding both deformation and breaking strength at 49 week, and then remained stable at 70 week of age. A decrease in shell weight and shell thickness without membranes was noted between 29 and 49 week of age and decreasing shell thickness with membrane after 49 week of age, resulting in reduced shell quality, most likely because a similar or smaller amount of shell is distributed to cover a larger egg, as has been suggested previously [3, 28]. Thus, our results show that the modern laying hen, represented by LSL and LB hybrids, produces eggs with a reduced shell quality quite early in the laying period, but that they are able to maintain this quality to at least 70 week of age. However, this pattern has not yet been determined for longer laying cycles [29].

During shell formation, the blood flow increases fivefold in the shell gland, to distribute the building material for shell formation. We found that the shell gland capillaries increased in number as the hens reached peak production at 29 week of age and stayed constant during the remainder of the production period. The number of capillaries that tested positive for CA activity also increased with age in both hybrids, but not until 49 week of age, i.e. half-way through the laying period. However, results from our previous experiments indicate that the number of capillaries with CA activity is lower in older hens and that hens induced to produce eggs with thinner shells show a reduction in capillaries with CA activity [20, 21]. This discrepancy between studies is difficult to explain, as the actual role of capillary CA in shell formation is not yet fully understood. It is known that a reduction in total shell gland CA affects the availability of both bicarbonate and calcium, since bicarbonate transport is to some extent coupled to calcium transport and inhibition of CA results in reduced calcium uptake by shell gland mucosa [18]. In tubular glands, we found membrane-bound staining for CA activity at all ages except 29 week and in the LB hens at 70 week, but the staining was patchy. However, gland density in the shell gland was lowest at 49 week, which may well have an effect on the bird’s ability to produce shell.

Bone strength of both femur and humerus was affected by age in our study and the first signs of weaker bone strength were detected in femur as early as 29 week of age. The strength of both femur and humerus was lowest at 49 week, i.e. in the middle of the hens’ production period. Progressive osteoporosis in modern laying hens, resulting in weaker bones, is initiated when the birds reach sexual maturity and continues throughout the laying period [30]. The theory is that the rise in oestrogen at the onset of sexual maturation results in a switch in bone formation from structural to the more dynamic medullary bone, which serves as a store for eggshell calcium (reviewed in [4]). The ability to store calcium in the skeleton decreases with age in laying hens, while egg production is still maintained at a high level and egg size increases, as seen in our study and many others. This eventually leads to a physiological imbalance when the total demand for calcium cannot be upheld and studies of bone fracture incidence show that it increases from 47 to 65 week of age [31].

Although bone strength of femur was more affected by age than that of humerus, the bone quality of the femur appeared to have recovered somewhat at 70 week of age. Housing system affects bone strength, and bone strength of the leg (tibia) generally improves in hens housed in conventional cages since the hen put a mechanical load on the leg when standing, but not the wing strength (radius) since the space for using the wing is very limited [32]. However, only the small movement onto a perch in a furnished cage improves the bone strength of the humerus [33]. Another possible explanation for the increase in mean bone strength found in older hens is an increase in periods when individual hens lay no eggs, which allows rebuilding of structural bone [5].

In the shell gland, both ERα and ERβ were found in the nuclei of tubular gland cells, in addition to weak cytosolic staining for ERβ. The nuclear staining was always the most prominent in both hybrids, regardless of age. Staining for ERα decreased at 49 week of age and then remained at that level throughout the hens’ production period. Hansen et al. [11] noted a decrease over time in ERα, but with the most dramatic drop somewhat later (71 week). The co-localisation of both ERs in the tubular glands suggests differences in functional roles between the two receptor subtypes, e.g. the receptors may counteract each other, as suggested for other tissues (reviewed in [16]). Regarding the function of tubular glands, the favoured hypothesis is that they provide the carbonate ions for shell formation [2, 34, 35]. This is supported by the presence of CA in these cells, as seen in this study and several others [20, 21]. However, other evidence suggests that tubular glands may have a dual function and provide at least part of the calcium ions needed [7, 36]. It is also known that secretion of HCO_3_^−^ and Ca^2+^ is partly coupled [37, 38]. Based on knowledge of ERα/ERβ activation in other tissues, it can therefore be speculated that activation of tubular gland ERα may stimulate secretion of Ca^2+^ and/or HCO_3_^−^ ions, while activation of ERβ-mediated processes may counteract this effect, or possibly fine-tune the concentrations of both ions for optimal shell formation. A decrease in ERα, but not ERβ, during the production period of laying hens would then cause an imbalance in this process, which may lie behind the reduction in shell quality. Note that in our study the reduced shell quality coincided in time with a reduction in tubular gland staining for ERα.

The nuclei of surface epithelial cells were stained for ERβ, but not for ERα, whereas ERα was found in the granules of the cytosol in the shell gland. The ERα staining pattern in surface epithelial cells was in agreement with earlier studies suggesting that granule production is highest during early shell formation, when both ciliated and non-ciliated cells contain large amounts of secretory granules between the apical surface and the nucleus [39]. However, in our study the presence of an egg in the oviduct (results not shown) was not correlated to granular staining intensity of ERα.

The non-ciliated cells of the surface epithelium have been implicated in the formation of the organic matrix of the shell and the final cuticle covering the finished eggshell [39]. These cells contained strong nuclear staining for ERβ, but no changes were detected during the production period that would indicate an effect on shell quality. Some nuclei of ciliated cells contained weak to moderate nuclear ERβ, which was most pronounced at 21 week compared with later in the production period. These cells contain the highest concentration of calcium [7, 36] and are likely to provide the bulk of these ions for shell formation.

Endothelial cells of both capillaries and larger blood vessels in the shell gland contained nuclear ERβ, which is in agreement with our earlier findings [13]. Interestingly, the staining intensity increased from 21 to 49 weeks in both hybrids and coincided with an increased number of capillaries with CA activity. It is known that CA can be up-regulated by oestrogen and in our previous study a dietary supplement of the phytoestrogen daidzein, a powerful inducer of ERβ-mediated processes, resulted in an increase of capillaries with CA in LB hybrids [13]. It is thus tempting to suggest that the regulation of capillary CA in the shell gland is mediated via ERβ [13].

Staining for ERβ was located in nuclei, but also in the cytosol of duodenal enterocytes. Oestrogen is involved in duodenal calcium absorption in a dual manner. In brief, oestrogen activates vitamin D_3_ to 1.25-dihydroxycholecalciferol (1.25D_3_) and up-regulates 1.25D_3_ receptors and synthesis of the calcium-binding protein D28K in intestinal mucosa, resulting in increased calcium absorption (reviewed in [40]). Oestrogen also affects calcium absorption in duodenum by acting directly on enterocytes, in a non-genomic manner independent of vitamin D [41, 42], the latter mechanism supporting the presence of extra-nuclear oestrogen receptors. Calcium absorption decreases with age in the laying hen and exogenous oestrogen boosts calcium uptake in duodenal tissue. However, no change in ERβ was detected during the production period in our study. The available data on oestrogen concentrations in laying hens over time are inconsistent [11, 43]. New evidence suggest that serum levels of 17β-estradiol decrease in older hens [10], which could lead to reduced absorption of calcium. However, the hens in that study were 12 weeks older than those in our experiment. Rather surprisingly, we found that the total level of calcium in plasma increased with age, suggesting that the bird’s ability to use calcium for eggshell and skeleton is affected by age in other ways.

As previously reported [13, 44], PMCA was found in the apical membrane of the tubular gland cells in the shell gland. The results indicated that shell gland PMCA is not involved in the reduced shell quality seen at 49 week in both hybrids. In duodenum, where the majority of calcium absorption takes place, PMCA was also located in the surface epithelial cells. According to previous research [42], PMCA in basolateral membranes of duodenal enterocytes participates in extrusion of absorbed calcium from enterocytes to the duodenal capillary network. In our study, PMCA was located in basolateral membranes of duodenal enterocytes, with the strongest staining at the top of the villi and weaker towards the villi base and crypt cells. In a previous study, calcium uptake was found to decrease with increasing age in three commercial hybrids [3], but our results on duodenal PMCA suggest that this specific calcium transporter is not involved in the decreased ability to absorb calcium. Interestingly, CA activity decreased dramatically in the duodenum at 49 week in our study and duodenal CA activity is reported to be lower in hens producing soft-shelled eggs [22]. Following the dip in CA activity at 49 week, it returned to the same level as in young birds when the hens reached 70 week. Even so, the results indicate reduced duodenal function at a time when egg production is still high. Despite the rather limited number of replicates in our study, significant age related effects on egg shell parameters and skeletal traits were to a large extent revealed. However, although major factors involved in these age related changes could be identified, it cannot be excluded that a larger study might have provided a stronger support for some of the suggested hypotheses.

## Conclusions

This study identified some key factors involved in shell formation and calcium absorption in parallel with changes in shell quality and bone strength in two layer hybrids. When the hens were around half-way through the production period, shell quality and bone strength had both deteriorated, but the birds were able to maintain the level thereafter until 70 week of age and bone strength even improved. Decreased gland density and a shift in the balance between ERα and ERβ in the shell gland, coinciding in time with a dramatic drop in duodenal CA activity, are suggested to be factors involved.

## Additional files

**Additional file 1.** Eggshell measurement; Carbonic anhydrase (CA) histochemistry; Immunohistochemistry; Image analysis and morphometric evaluation.

**Additional file 2.** Immunohistochemical evaluation of ERβ in the shell gland; Immunohistochemical evaluation of PMCA in duodenum.

## Abbreviations

CA: carbonic anhydrase
ERα: oestrogen receptor alpha
ERβ: oestrogen receptor beta
LB: Lohmann Brown
LSL: Lohmann Selected Leghorn
PBS: phosphate-buffered saline
PMCA: plasma membrane Ca^2+^ ATPase
