# Supplementary material for: Age-related changes in the shell gland and duodenum in relation to shell quality and bone strength in commercial laying hen hybrids
Source: Acta Vet Scand. 2019 Mar 12;61:14. doi: 10.1186/s13028-019-0449-1 (PMC6417018; doi:10.1186/s13028-019-0449-1)
Supplement: Supplementary file 1 — Additional file 1. Eggshell measurement; Carbonic anhydrase (CA) histochemistry; Immunohistochemistry; Image analysis and morphometric evaluation. [file 13028_2019_449_MOESM1_ESM.pdf]

Additional file 1

Eggshell measurements

Shell deformation was calculated from the average value of measurements on three marking points on the equator of the egg, after a load of 1,000 g was applied on the egg (The Canadian Egg Shell Tester, Otal Precision Company Ltd, Ottawa Ontario, Canada K1G3N3). The shell breaking strength was recorded for each egg using the same instrument. The shell pieces were clean of albumen and yolk with distilled water and dried over night at 120°C. Shell weight including shell membranes was recorded and shell thickness was measured using a digital micrometer (Mitutoyo Absolute, No. 7360; Mitutoyo Corp., Stockholm, Sweden) at the three marked points along the equator. Shell membranes were removed by boiling the shells in 2.5% (w/v) NaOH for 8 min, rinsing in distilled water and drying overnight at 120°C. Shell thickness was then measured without membranes according to the same procedure as above.

CA histochemistry

Sections (2 µm) of resin embedded tissues were cut on a microtome (Leica RM 2165, Leica Instruments, Germany) using glass knives. The sections were incubated for 6 min floating on the incubation medium containing 3.5 mM $CoSO_4$, 53 mM H_2SO_4, 11.7 mM KH_2PO_4 and 157 mM $NaHCO_3$. After incubation the sections were rinsed on 0.67 mM phosphate buffer (pH 5.9), transferred to 0.5% (v/v) $(NH_4)_2S$, and finally rinsed on two successive baths of distilled water. The incubation procedure results in a black precipitate of cobalt sulphide at sites with CA activity. Before mounting, some of the sections were counter-stained with azure blue. Neighbouring sections were stained with hematoxylin-eosin for conventional histology. The specificity of the reaction was checked using the CA inhibitor acetazolamide. Sections were first pre-incubated on a 10 µM solution of acetazolamide for 30 min and then incubated as above but with an incubation medium containing 10 µM inhibitor.

Immunohistochemistry

The paraffin embedded tissues were cut into 4µm thick sections and mounted on Superfrost Plus Gold slides, (Menzel-Glaser, Braunschweig, Germany), deparaffinized in xylene and rehydrated in graded alcohol. The sections were rinsed in PBS buffer after each step in the following procedure. Antigen retrieval was performed by pressure heating for 20 minutes in a pressure-boiler (21100 Retriever, Histolab Products AB, Gothenburg, Sweden) using 0.01 M sodium citric buffer (pH 6.0). Endogenous peroxidase activity was blocked with 3% hydrogen peroxide. All sections were treated with normal serum provided by the kit for the secondary antibody used in the respective assay (see below).

The sections were incubated in dark with primary antibodies at room temperature in two hours for ERβ and PMCA, and at +4 °C over night for ERα. ERα was detected using a rabbit anti-ERα (clone 60C, Millipore, USA) diluted 1:50, ERβ was detected using a mouse monoclonal antibody (MCA 1974ST, Serotec, Düsseldorf, Germany) diluted 1:20 and PMCA was detected using a mouse monoclonal antibody (5F10 ab2825, Abcam 330, Cambridge Science Park, Cambridge, CB4 0FL, UK) diluted 1:1000.

The secondary antibody used in the assays for ERα, ERβ and PMCA was a peroxidase labeled anti-rabbit or anti-mouse IgG (Cat. No. MP-7401 and MP-7402, ImmPRESS reagent kit, Vector Laboratories, Burlingame, CA, USA). All sections were treated with the chromogen

3.3´-diaminobenzidine tetrahydrochloride (DAB-safe, Saveen Biotech, Malmö, Sweden) to which H_2O_2 was added to visualize the bound enzyme activity as a brown color.

Negative controls were run by omitting the primary antibodies and by replacing the primary antibody with non-immune serum from mouse (sc-2025) in the ERβ and PMCA analyses, and from rabbit (sc-2027) in the ERα analyses (Santa Cruz Biotechnology, Inc., Santa Cruz, CA. 95060 USA). Sections from rooster testis and epididymis were used as positive control for ERα and ERβ and pig ovary and uterus for ERβ. All sections were mounted with Pertex (Histolab products AB, Gothenburg, Sweden).

Image analysis and morphometric evaluation

For evaluation of CA activity in the shell gland one image from each top of five consecutive mucosal folds, containing mucosal surface epithelium and sub-epithelial tubular glands, were analysed, i.e. five images/section and hen. Only mucosal folds attached to the underlying submucosal layer were chosen for analysis. The total number of capillaries/mm^2 and the number of capillaries positive for CA/mm^2 were recorded using an image analysis software (Elcipse Net, version 1.20, Developed by Laboratory Imaging, Prague, Czech Republic). Capillaries were counted only when their entire circumference was located within the picture frame. A capillary was considered CA positive when more than half of its circumference showed CA activity (black staining).

For evaluation of CA activity in the duodenum, membrane-bound and cytosolic activity were noted and the intensity of the staining was scored on a scale from 1-3. Score 1: staining weak or absent, score 2: intermediate and score 3: strong staining.

The height of mucosa and depth of crypts of Lieberkühn in the duodenum were measured in five fields/section. The height of mucosa was measured from muscularis mucosa to the top of the villi and the depth of crypts of Lieberkühn was measured from muscularis mucosa to the base of the villi, a mean was calculated for each bird.
