# Supplementary material for: Age-related changes in the shell gland and duodenum in relation to shell quality and bone strength in commercial laying hen hybrids
Source: Acta Vet Scand. 2019 Mar 12;61:14. doi: 10.1186/s13028-019-0449-1 (PMC6417018; doi:10.1186/s13028-019-0449-1)
Supplement: Supplementary file 2 — Additional file 2. Immunohistochemical evaluation of ERβ in the shell gland; Immunohistochemical evaluation of PMCA in duodenum. [file 13028_2019_449_MOESM2_ESM.pdf]

Additional file 2

Immunohistochemical evaluation of ERβ in the shell gland.

In the shell gland the localization was described and the staining of each structure on one slide/bird was scored for intensity on a scale from 0-3, where zero corresponds to no staining and three representing strong staining. Morphometric staining intensity were analysed by likelihood Ratio Chi-Square test using SAS®. The statistical models included the fixed effects of hybrid (n=20) and age (n=10). Each hen was treated as an experimental unit, i e five replicates per hybrid and age. The tubular gland cells showed strong nuclear staining and weak cytosolic staining. The cytosolic staining was strongest at 29 wk of age and differed compared to the staining at age 49 wk (P= 0.004) and between the age of 21 wk (P= 0.04) and 29 wk and between the age 29 wk and 70 wk (P= 0.047).

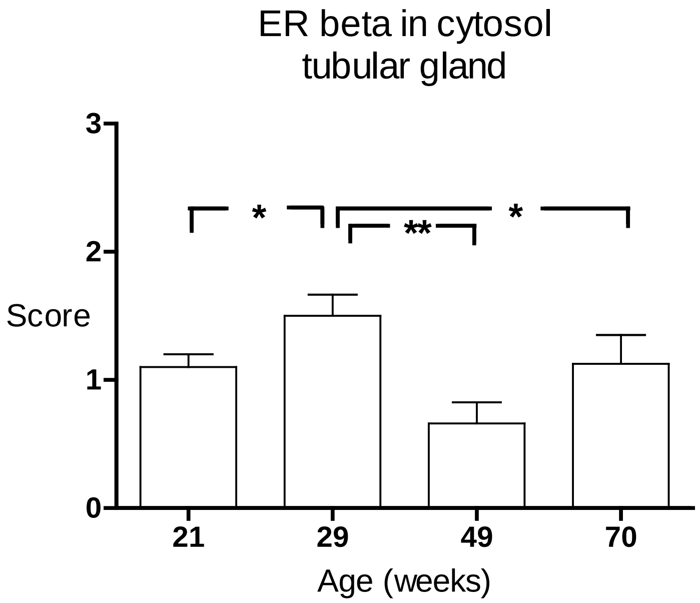

Intensity scoring of oestrogen receptor β (ERβ) in shell gland mucosal fold of LB and LSL hens (n=20) at four different ages during a production period. Score 0: no staining. Score 1: weak staining. Score 2: intermediate staining. Score 3: strong staining.

Immunohistochemical evaluation of PMCA in duodenum.

In duodenum staining for PMCA was found in the baso-lateral membranes of the surface epithelial cells. The staining intensity was strongest at the top of the villi and gradually decreased towards the base. The staining intensity of the villi was not affected by age or hybrid.

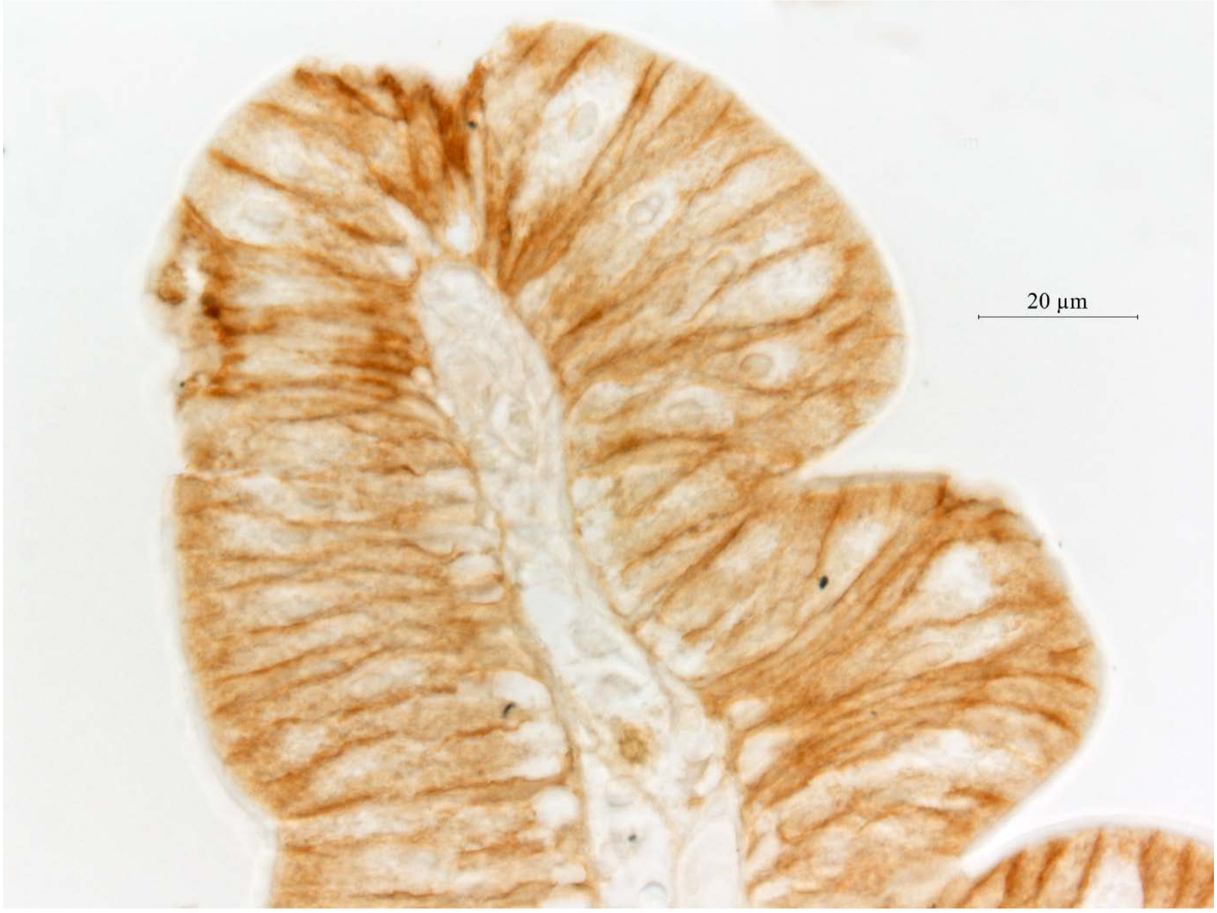

Plasma membrane Ca^{2+} ATPase (PMCA) immunolocalisation. Duodenum of 29 week-old Lohmann Brown (LB) hen. Strong staining in the baso-lateral membrane of the surface epithelial cells, bar = 20µm.
